# Genetic variation associated with healthy traits and environmental conditions in *Vaccinium vitis-idaea*

**DOI:** 10.1186/s12864-017-4396-9

**Published:** 2018-01-02

**Authors:** Zobayer Alam, Julissa Roncal, Lourdes Peña-Castillo

**Affiliations:** 1 Department of Biology, Memorial University of Newfoundland, St. John’s, NL A1B 3X9 Canada; 2 Department of Computer Science, Memorial University of Newfoundland, St. John’s, NL A1B 3X5 Canada

**Keywords:** Antioxidant capacity, Environmental adaptation, Functional annotation, Genetic diversity, Genotyping-by-sequencing, Lingonberry, Phenolic content, Single nucleotide polymorphism

## Abstract

**Background:**

Lingonberry (*Vaccinium vitis-idaea* L*.*), one of the least studied fruit crops in the *Ericaceae* family, has a dramatically increased worldwide demand due to its numerous health benefits. Genetic markers can facilitate the selection of berries with desirable climatic adaptations, agronomic and nutritious characteristics to improve cultivation programs. However, no genomic resources are available for this species.

**Results:**

We used Genotyping-by-Sequencing (GBS) to analyze the genetic variation of 56 lingonberry samples from across Newfoundland and Labrador, Canada. To elucidate a potential adaptation to environmental conditions we searched for genotype-environment associations by applying three distinct approaches to screen the identified single nucleotide polymorphisms (SNPs) for correlation with six environmental variables. We also searched for an association between the identified SNPs and two phenotypic traits: the total phenolic content (TPC) and antioxidant capacity (AC) of fruit. We identified 1586 high-quality putative SNPs using the UNEAK pipeline available in TASSEL. We found 132 SNPs likely associated with at least one of the environmental or phenotypic variables. To obtain insights on the function of the genomic sequences containing the SNPs likely to be associated with the environmental or phenotypic variables, we performed a sequence-based functional annotation and identified homologous protein-coding sequences with functional roles related to abiotic stress response, pathogen defense, RNA metabolism, and, most interestingly, phenolic compound biosynthesis.

**Conclusions:**

The putative SNPs discovered are the first genomic resource for lingonberry. This resource might prove useful in high-density quantitative trait locus analysis, and association mapping. The identified candidate genes containing the SNPs need further studies on their potential role in local adaptation of lingonberry. Altogether, the present study provides new resources that can be used to breed for desirable traits in lingonberry.

**Electronic supplementary material:**

The online version of this article (10.1186/s12864-017-4396-9) contains supplementary material, which is available to authorized users.

## Background

*Vaccinium vitis-idaea* L. commonly known as lingonberry, is a perennial, evergreen dwarf shrub, which belongs to the Ericaceae family, that has high breeding potential for leaf and fruit quality traits such as high concentration of healthy bioactive compounds. *Vaccinium* is a good source of pharmaceutical ingredients because it is rich in phenolic phytochemicals (e.g. flavonoids, phenolic acids), which have proven to reduce the risk of cancer development [1], hepatitis C [2], cardiovascular disorders [3], diabetes [4], and urinary tract infections [5]. Among the commonly cultivated “berry” fruit such as cranberry, strawberry, raspberry, and blueberry, *V. vitis-idaea* ranks high in antioxidant capacity as conferred by phenolic compounds [6, 7]. Despite its long cultivation history in Scandinavian countries, in North America, breeding is at its developmental stage. Interestingly, the North American *V. vitis-idaea* subsp. *minus* (Lodd) Hult. has a higher anthocyanin content and antioxidant capacity than the Eurasian *V. vitis-idaea* subsp. *vitis-idaea* (L.) Britton [8, 9], increasing the interest to develop a breeding and commercialization program for the North American subspecies.

While it is well known that environmental conditions can affect the concentration of phenolic content and antioxidant capacity in *V. vitis-idaea* (e.g. [9, 10]), very few studies have characterized the genetic diversity of wild populations in North America [8, 11, 12]. Neither the complete nuclear genome nor the plastome have been sequenced for this species, and no single nucleotide polymorphisms (SNP) or expressed sequence tags (EST) are available as genetic resources. Genetic markers are therefore needed to facilitate the selection of wild populations with desirable climatic adaptations, agronomic and nutritious characteristics.

In the face of environmental change, it is becoming more important to understand the genetic variation that results from selection to different environmental growing conditions. For several crops, common garden and transplant experiments have revealed strong adaptive clines in growth, phenology traits and physiological responses to abiotic conditions, and sometimes the adaptive genetic variation underlying these differences were revealed (e.g. [13, 14]). Phenolic compound biosynthesis genes have been identified in different plants especially as regulated by the excess or deficiency of the phenylalanine ammonia lyase (*PAL*), an enzyme that catalyzes the first step of the phenylpropanoid pathway, which produces precursors to several important secondary metabolites [15]. For example, the expression of nine phenolic acid biosynthesis pathway genes was closely related to phenolic acids accumulation during grain filling in wheat [16]. Polymorphism of genes encoding isoflavone synthase, an enzyme involved in the phenylpropanoid pathway, was associated with isoflavone concentrations in soybean seeds [17].

Genotyping-by-sequencing (GBS, [18]) is a practical and inexpensive method for high-throughput SNP discovery and genotyping through next-generation sequencing (NGS) technologies. This approach conducts a multiplex sequencing of restriction site-associated DNA, and has been successfully used in crops for numerous applications like quantitative trait loci marker identification [19], characterization of germplasm diversity and conservation [20]. In *Vaccinium*, this technique successfully identified markers for root architecture trait breeding [21].

Knowledge of the genetic basis of phenotypic variation and adaptation to local environmental conditions is necessary for the success of a nutrition-oriented breeding program for lingonberry. Thus, the goals of the current study were: 1) to use the GBS approach for high-throughput identification of intraspecific putative SNPs in *V. vitis-idaea* subsp. *minus,* 2) to assess whether or not identified putative SNPs were correlated with environmental or phenotypic variables, as a potential signature of adaptation, and 3) to identify and functionally annotate protein-coding genomic sequences containing SNPs exhibiting correlation with environmental or phenotypic variables, as an insight to their potential role in local adaptation.

## Methods

**Fig. 1:**
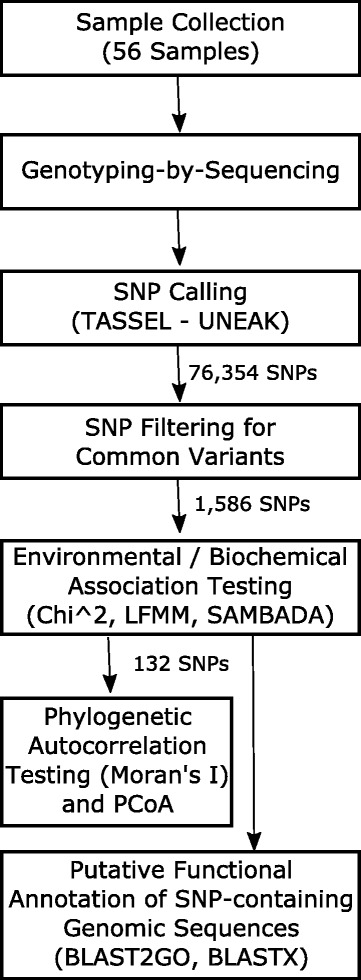
Workflow followed in this study from sample collection to functional annotation of putative SNPs for *Vaccinium vitis-idaea subsp. minus*

Figure 1 illustrates the workflow followed in this study. In the following subsections we describe each step in detail.

### Wild site selection, environmental variables, and plant material collection

Although there is no cultivation practiced in Canada for lingonberry, the province of Newfoundland and Labrador (NL) is the top wild lingonberry fruit producer in Canada with a harvest range of 40 to 500 tons per year [22]. In August 2014, we collected lingonberry adult leaf samples from 56 wild sites distributed across NL. Leaves from one stem were collected per site and stored in silica gel. To elucidate the genetic variation across environmental conditions, we considered the following six variables: mean annual temperature (MAT), mean summer temperature (MST), mean annual precipitation (MAP), mean annual runoff (MAR), surface water pH (SWp), and surface water sensitivity to acid rain (SWS). Using the most updated information from the Water Resources Division of the Government of Newfoundland and Labrador [23] we partitioned the province into categories within these six variables, thus we worked with discrete environmental variables. The environmental characteristics associated with each collection site, a map, and their geographic coordinates were disclosed in Alam et al. [10] and in Additional file 1: Table S1. A voucher specimen was deposited in the NFLD herbarium (JR500).

### GBS assay and SNP calling

We extracted total genomic DNA from silica gel dried leaf samples using the DNeasy Plant Mini Kit (Qiagen). The quality and quantity of extracted DNA was evaluated using a Qubit 2.0 Fluorometer (Life Technologies) and a NanoDrop 1000 spectrophotometer (Thermo Fisher Scientific). An estimated quantity of 100 ng of total genomic DNA was used to prepare each library, in a volume of 10 μL of EB buffer. Reduced representation GBS libraries were prepared for each DNA sample using a double-digest restriction protocol with enzymes *Pst*I and *Msp*I as described in Poland et al. [24] (University of Laval), and sequenced (single-end reads) in one lane using Illumina HiSeq 2500 (McGill University-Génome Québec Innovation Center).

The raw Illumina data were processed following the protocol of Poland et al. [24]. Raw DNA read quality was investigated using FASTQC (Banraham Bioinformatics, Cambridge, England). We used the TASSEL-UNEAK v.3.0 network-based de novo SNP discovery pipeline without a reference genome [25] to identify accurate and high quality SNPs. In the TASSEL-UNEAK pipeline, the multiplexed reads were sorted into unique sequence tags by compiling exactly matching reads with at least ≥3X read depth. To reduce the rare or singleton tag from sequencing error, the created taglist (identical reads classified as a tag) was filtered with ≥5X tag depth. Pairwise alignments were conducted between tag pairs and tag pairs with 1 bp mismatch were considered as putative SNPs. An homology network was constructed by joining all of the tags that differed by a single base, and then a network filter was applied to find reciprocal tag pairs [25] based on a sequencing error tolerance rate (ETR) of 0.05. The created HapMap file was then filtered using the default minor allele frequency (MAF) of 0.05, and a maximum allele frequency of 0.5 to call SNPs. Using a custom R script, putative SNPs were further filtered for minimum coverage threshold at 3X, minimum minor allele frequency at 0.30, and call rate higher than 50%. A minimum MAF of 0.30 was selected so that the putative SNPs were highly polymorphic and produced clearly separated genotypic clusters [26].

### Putative SNPs associated with environmental and phenotypic variables

To identify some of the loci putatively responsible for adaptive differences among individuals, we applied three methods to test for correlations between allele frequencies and the environmental or phenotypic variables. These methods were Chi-square test [27], Latent Factor Mixed Models (LFMM, [28]), and a logistic regression-based approach (Samβada, [29]). LFMM estimates the influence of population structure on allele frequencies by introducing unobserved variables as latent factors [28]. Samβada uses logistic regressions to model the probability of observing a SNP genotype given the environmental conditions at the sampling location [29]. Fruit total phenolic content (TPC) and antioxidant capacity (AC) for *V. vitis-idaea* sampled in this study were analyzed previously in Alam et al. [10]. To be able to apply Chi-square test with the phenotypic variables, samples were categorized into three groups according to their TPC as low (< 650 mg GAE/100 g FW), medium (650–850 mg GAE/100 g FW), and high (> 850 mg GAE/100 g FW); and samples were categorized into two groups according to their AC as low (<650 mg TE/100 g FW), and high (> 650 mg TE/100 g FW). For Chi-square test, Monte Carlo simulations (10,000 replicates) were performed. To run LFMM, the function lfmm available in the R package LEA (version 1.6.0) was executed with two as the number of latent factors, ten repetitions, and 20 k total and 10 k burnin iterations in the Gibbs Sampling algorithm. All other parameters were set to their default values. Samβada (version 0.5.3) was executed with DIMMAX equal to 3, SAVETYPE set to “end best”, SPATIAL set to “Longitude Latitude SPHERICAL NEAREST 2” and AUTOCORR “BOTH BOTH 1000”. A *p*-value of 0.01 was used to call significant associations identified by each approach. Only putative SNPs identified as significantly associated with at least one environmental or phenotypic variable by at least two approaches were used for further analyses.

### Phylogenetic autocorrelation with environmental/phenotypic variables and principal coordinates analysis (PCoA)

To gather further support to the potential association of the putative SNPs deemed to be significantly associated with each variable, we conducted a Moran’s I test and PCoA. For each variable, the pairwise genetic distances between samples taking into account only those SNPs found to be significantly associated with the given variable were calculated using the function dist.gene available in the R package ‘ape’ [30]. To calculate the Moran’s I autocorrelation coefficient, samples were clustered using the BIONJ algorithm using the function bionj in ‘ape’, then their pairwise cophenetic distances were computed from the pairwise genetic distances using the ape’s function cophenetic, and finally ape’s function Moran.I was executed. A cutoff *p*-value of <0.05 was used to determine whether a given phenotypic or environmental variable had a significant phylogenetic autocorrelation. If the observed Moran’s I value is significantly greater than the expected I value, then it is considered as positively autocorrelated, while if the observed Moran’s I value is smaller than the expected I value, then it is negatively autocorrelated [30]. To further observe the discrimination of samples according to health traits and environmental variables as revealed by their associated SNPs, we conducted a PCoA using the function dudi.pco available in the R package adegenet (version 2.0.1) [31]. To perform PCoA, the pairwise genetic distances were converted to Euclidean distances using the adegenet’s function quasieuclid. PCoA plots were created using the adegenet’s function s.class and add.scatter.eig.

Morales [32] evaluated the genetic similarities of these *Vaccinium vitis-idaea* samples using the consensus sequences of DNA reads towards the reconstruction of a Bayesian phylogenetic tree. He found three genetic groups in the tree showing a geographic structure according to ecoregion and temperature, with genetically close individuals also being geographically close.

### Putative function of reads with SNPs

To obtain a putative functional annotation of the protein-coding genomic regions were putative SNPs were found, we performed BLASTX (version 2.6.1+) searches against the NCBI Non-Redundant (NR) protein database using BLAST2GO [33] (version 4.1.7) with an E-value <0.001. The first step in BLAST2GO was to find proteins with the highest DNA sequence similarity to our query DNA sequence and retrieve them for further analysis. The next step was to provide a functional analysis of proteins by classifying them into families and predicting domains using the InterPro function of BLAST2GO. Mapping was performed to retrieve gene ontology (GO) terms associated to the hits obtained after the BLASTX search. GO terms assigned were restricted to the plant slim GO subset. Additionally, we searched for similar sequences in RNAcentral [34] (release 7), a comprehensive database of non-coding RNA sequences, using nhmmer [35] with an E-value <0.001 and all other parameters set to default values.

## Results

### Library sequencing, SNP discovery and selection

Sequencing of a 56-plex GBS library yielded about 142 million barcoded reads 100 bp long, corresponding to an average of 2,53 million reads per sample and ranging from 0,94 to 6,02 million reads per sample. GBS data is available at the NCBI with the BioProject accession number PRJNA377297. A quality analysis using FastQC reported an average Phred quality score of 34, and % GC content of 53. The TASSEL-UNEAK pipeline yielded 76,354 SNP loci putatively located at a single locus, with MAF of 0.05. Using a custom R script, a set of 1586 SNPs with a read depth coverage of at least 3X, genotyped in at least half the samples, and a minor allele frequency greater than 0.30, was identified and selected for further genetic characterization analysis. On average, there was 13.4 ± 7.3% of missing data per sample across the selected dataset of 1586 SNP loci.

### Putative SNPs associated with phenotypic and environmental variables

**Fig. 2:**
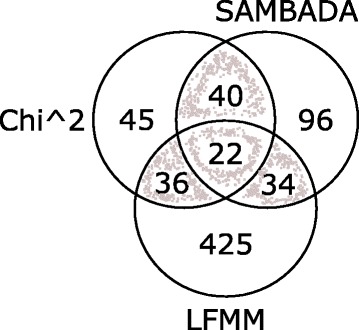
Venn diagram showing the total number of putative SNPs significantly associated with at least one environmental or phenotypic variable by three approaches for detecting loci under selection: chi-square, LFMM and Samβada. A *p*-value of 0.01 was used to call significant associations identified by each approach. The 132 SNPs on the intersections (shaded area) were considered for further analysis

**Fig. 3:**
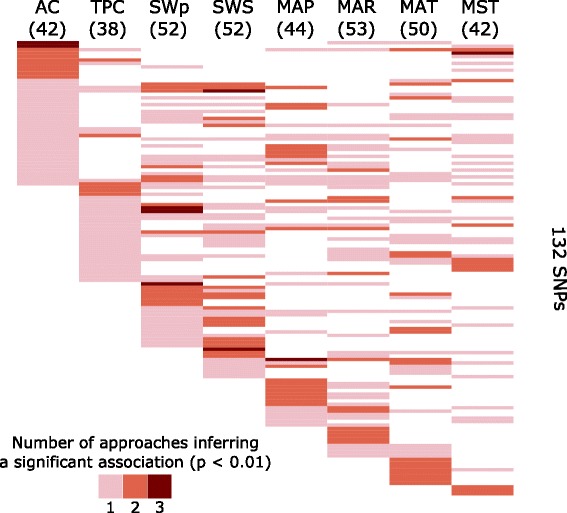
Comparison of putative SNPs significantly associated with each environmental and phenotypic variable. Each row corresponds to a putative SNP that was significantly associated with at least one variable by at least two approaches (Fig. 2). Numbers in brackets under the variable identifiers indicate the total number of SNPs associated to each variable. Color bars indicate the number of approaches supporting each SNP – variable association. AC = antioxidant capacity, TPC = total phenolic content, SWp = surface water pH, SWS = surface water sensitivity to acid rain, MAP = mean annual precipitation, MAR = mean annual runoff, MAT = mean annual temperature, MST = mean summer temperature

The Chi-square test, LFMM, and Samβada identified 143, 517, and 192 SNPs respectively, significantly associated with phenotypic and/or environmental variables (Fig. 2). We found 132 SNPs significantly associated with at least one variable using at least two approaches. Of these 132, the maximum number of associated SNPs (53) was identified for the variable MAR, and the minimum (38) for the variable TPC (Fig. 3 and Additional file 1: Table S2). 102 (or 82%) of the 132 SNPs were correlated with more than one variable (Fig. 3 and Additional file 1: Table S3). On average, there were 17.4 ± 5.5 SNPs shared between each pair of variables. Surface water variables (SWS and SWp) shared the highest number of SNPs (37), while the phenotypic variables (TPC and AC) shared the lowest number of SNPs (10). On average, 3 ± 2.1 SNPs were uniquely associated to one variable. The variable MAT has the largest number of SNPs (7) exclusively associated to it. The variables TPC and SWS have both only one SNP exclusively associated to them.

### Phylogenetic autocorrelation with environmental/phenotypic variables and PCoA

**Fig. 4:**
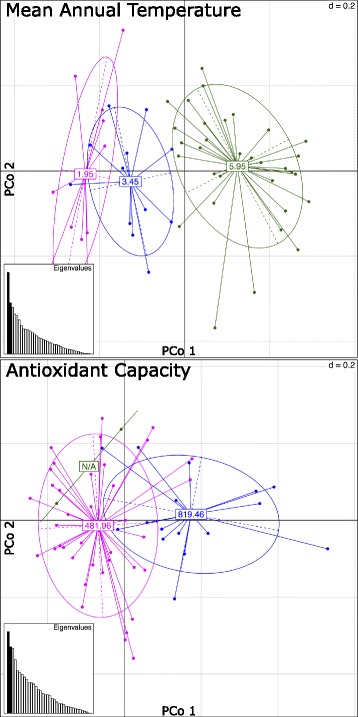
Principal Coordinates Analysis (PCoA) plots for two variables: MAT on top and AC on bottom. Dots represent individual samples and are colored based on the values of the corresponding environmental or phenotypic variable. Numbers at center of clusters are mean values for each group. X and Y-axes represent first and second multivariate coordinate (PCo1 and PCo2), respectively. d = 0.2 represents the mesh of the grid. Only the 50 and 42 SNPs associated with MAT and AC, respectively, were used to obtain the genetic distance between samples, and the clustering of the samples based on the variable values indicate that these SNPs are indeed correlated with these variables. The bar plot on the bottom left corner shows the eigenvalues

Moran’s *I* statistics indicated a statistically significant positive phylogenetic autocorrelation for all environmental and phenotypic variables supporting the results of the Chi-square, LFMM, and Samβada. Moran’s *I* expected and observed values, and *p*-values are shown in Additional file 1: Table S4. PCoA plots showed that individuals collected from the same environmental condition or showing similar phenotype were genetically more similar when considering the SNPs associated with the corresponding variable. Fig. 4 illustrates this for MAT and AC. Principal coordinate values for each sample appear in Additional file 1: Table S5 and S6. PCoA plots for the remaining variables are provided in Additional file 2.

### Putative function of reads with SNPs

**Fig. 5:**
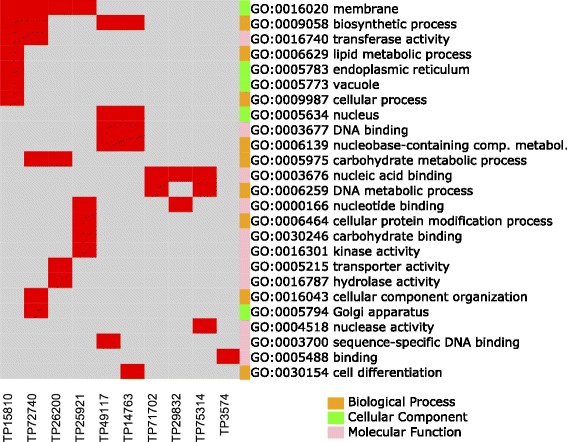
Distribution of gene ontology annotations for *Vaccinium vitis-idaea subsp. minus* genomic sequences obtained by BLAST2GO. Red bars indicate that a particular SNP-containing gene is annotated with the given GO term. The color bars to the left of the GO term identifiers indicate the category to which that particular GO term belongs

To identify protein coding genes harbouring the 132 SNPs associated with environmental and phenotypic variables, the corresponding SNP-containing reads were used as query for BLASTX searches to the NCBI NR protein database. BLAST2GO was used for assigning a functional annotation based on sequence homologies. Most of *V. vitis-idaea* sequences containing the 132 SNPs had no significant sequence alignment or hits in the NCBI NR database. The BLAST2GO annotation produced GO term assignments for 10 sequences out of the 14 genomic sequences with homologous proteins. These 14 genomic sequences with homologous proteins are listed in Table 1, and Fig. 5 indicates the GO term assignments obtained.

**Table 1:** Functional annotations of 14 SNP-bearing sequences significantly associated with phenotypic and environmental variables

SNP ID	Associated Environmental Variable	BLAST2GO Functional Annotation	E-value	% Identity
TP3574	MAR	Pentatricopeptide repeat-containing mitochondrial	1.79042E-4	96
TP14763	TPC	Transcription factor (contains a MYB DNA-binding domain)	3.94276E-5	93
TP15810	SWp, MAP, MAR	Phospholipid:diacylglycerol acyltransferase 1-like	7.36315E-5	100
TP25921	TPC, SWp, SWS, MAR, MAT, MST	Leaf rust 10 disease-resistance locus receptor-like protein kinase-like	1.57258E-5	86
TP26200	TPC, SWS	Glucose-6-phosphate phosphate translocator chloroplastic-like	1.28322E-5	100
TP29832	MAP, AC	RNA-binding CP31B chloroplastic	3.68209E-4	100
TP45867	SWp, SWS	DEAD-box ATP-dependent RNA helicase 37-like	6.29099E-4	100
TP49117	MAT	Calmodulin-binding transcription activator 4 isoform X3	1.35641E-6	95
TP60073	MAT	Pre-mRNA-splicing factor ATP-dependent RNA helicase DEAH1-like	6.21354E-4	100
TP69971	MAP, SWp, SWS	NAD(P) FAD-dependent oxidoreductase	3.22832E-5	100
TP71702	MAR, SWS, TPC	Unnamed protein product, partial	4.30509E-4	88.33
TP72119	AC	Uncharacterized protein	3.3681E-5	100
TP72740	MAT, SWS	Galacturonosyltransferase	3.34427E-6	100
TP75314	MST, SWp, SWS	Hypothetical protein VITISV_032719	2.16821E-4	93

Homologous sequences were retrieved by BLAST2GO using blastX with an E-value <0.001

The gene ontology encompasses terms describing genes and gene product roles in cells [36]. These terms are in three categories: 1) biological process – referring to the biological objective of the genes or gene products; 2) cellular component – referring to the place in the cell where the gene product is active; and 3) molecular function – referring to the biochemical activity of the genes or gene products. In the biological process category, the most common GO term was biosynthetic process (4 proteins). The cellular component was dominated by membrane (4 proteins). The molecular function category was comprised of protein coding genes involved in compound binding, transferase activity, and DNA-binding. This GO analysis suggested that *V. vitis-idaea* SNP-containing sequences code for diverse proteins involved in structural, regulatory, and metabolic processes (Fig. 5).

As there were only 14 genomic sequences with homologous proteins, we additionally searched for homologous non-coding RNAs to the sequences containing the 132 SNPs in RNACentral. Two SNP-containing sequences have a homologous non-coding RNA: SNP ID TP14976 has significant sequence similiarity to *Hevea brasiliensis* miscellaneous RNA URS0000A1EE58 (E-value = 8.3E-6), and SNP ID TP52730 has significant sequence similarity to *Hevea brasiliensis* miscellaneous RNA URS0000A2A2E6 (E-value = 3.9E-5). Both homologous non-coding RNAs lack functional annotation.

## Discussion

### Putative SNPs associated with phenotypic and environmental factors

Different methods to identify correlations between environmental variables and allele frequencies have their own advantages and weaknesses [37], and various factors can confound inferences of local adaptation [38]. To be more confident about our findings, we used three complementary approaches (i.e., Chi-square, LFMM, and Samβada) based on different principles. We interpreted SNPs found to be significantly associated with an environmental or phenotypic variable by only one of the approaches as spurious correlations (false positives); although, by doing this we are increasing the number of false negatives. As environmental variables may be highly correlated, approaches were not required to agree on the same associated variable (Fig. 3). The large proportion (82%) of the 132 SNPs that were associated to more than one variable suggests that indeed variables are correlated.

Our finding of only ten putative SNPs associated with both TPC and AC suggests that the genetic basis for the differential TPC and AC of fruit is not the same, and that the AC of fruit extracts depends not only on the specific phenolic compound profile but also on other non-phenolic molecules. This lack of positive correlation between TPC and AC has previously been reported in several studies [10, 39, 40]. Our understanding of how exactly phenolic compounds contribute to the antioxidant activity of different plant species is still limited. The contribution of each individual phenolic compound to the total AC may vary [41], thus variation in the specific phenolic compound profile can affect the overall AC. For example, the AC of different flavonoids depends on the number and position of hydroxyl substitutions on their backbone structure [42], and glycosylation might result in lower AC [43]. Plants possess different enzymatic (e.g. superoxide dismutase, catalase, ascorbate peroxidase) and non-enzymatic (e.g. glutathione, phenolic compounds, alkaloids, non-protein amino acids, and α-tocopherols) antioxidant defense systems, which protect them from oxidative damage by scavenging reactive oxygen species [44].

### Potential functions of putative SNPs associated with environmental and phenotypic variables

Among the 132 putative SNPs that were found associated with environmental or phenotypic variables, only 14 SNP-containing DNA sequences (10.6%) yielded hits in the NCBI NR protein database. These results suggest that most of these putative SNPs were located in unknown proteins (i.e., proteins not yet in the database) or in non-coding genomic regions. We discuss the gene identity and putative function of each of the 14 SNP-containing sequences as revealed from BLAST2GO and the literature search except for SNPs ID TP72119, TP75314, and TP71702 because their functional annotation rendered a protein of unknown function (Table 1).

The most significant blastx hits (95% identical) for SNP ID TP49117 corresponded to calmodulin-binding transcription activator 4 (CAMTA 4). In plants, the calcium ion is the most ubiquitous secondary messenger, participating in the transduction of various signals such as cold, wind, touch, UV light, pathogen attack, and phytohormones [45]. The mechanisms of Ca2 + −dependent transcription regulation include various signal transducers such as CAMTAs. It is hypothesized that CAMTAs are involved in biotic and abiotic stress responses by modulating hormone signalling [46]. More specifically for our study, CAMTA 4 positively regulates auxin transport and homeostasis, upregulates specific toxin metabolic processes, and together with CAMTA 5 and 6 regulates more than 1000 downstream genes linking environmental cues to growth responses [47]. This SNP-containing region might provide insight on CAMTA-based regulation of cold response, since it was associated with MAT.

The most significant blastx hits yielded from the sequence containing SNP ID TP72740 were annotated as probable galacturonosyltransferase 6 (GAUT). GAUTs are among the enzymes responsible for pectin and xylan biosynthesis in cells walls and seed testa [48]. Pectins are highly heterogenous polymers that control cell wall porosity, cell adhesion, plant defense, and resistance to extracellular freezing [49, 50]. Cold acclimation in *Brassica napus* has been associated with increased cell wall thickness, modifications in leaf stiffness, cell wall and pectin contents, pectin methylesterase activity, and the low-methylated pectin content [51–53]. Therefore, this correlated SNP with MAT and SWS is worth further evaluation especially because few pectin biosynthesis genes have been identified [48].

A protein similar to a receptor-like protein kinase (RLK) was annotated for an homologous sequence to SNP ID TP25921. In *Arabidopsis*, the RLK gene family contains more than 600 members, and it is the largest gene family in plants [54]. Many RLKs are known to be involved in several biological functions, including the regulation of plant development, growth, environmental stress response, or pathogen defense [55, 56]. Among the abiotic stress factors that induce RLKs are cold, drought, salt, toxic metals, and mineral deficiency [57]. Since this SNP was associated with TPC and several environmental variables (SWS, SWp, MAR, MAT, MST), and the fact that few RLKs have an experimentally verified function, we cannot hypothesize on a potential adaptive role of this particular locus until further biochemical and functional characterization are conducted.

The most significant blastx hits for SNP ID TP26200 were 100% identical to a segment (21 aminoacids) of glucose-6-phosphate/phosphate translocator (GPT). GPTs are involved in delivery of glucose 6-phosphate to plastids from non-green tissues as carbon sources for starch biosynthesis and/or to the oxidative pentose phosphate pathway [58]. Of the two GPT genes in *A. thaliana,* expression of GPT2 seems highly variable and sensitive to impaired carbon metabolism [59], sugar-induced senescence [60], phosphate starvation [61], and increases in carbon fixation due to increased light [62]. This latter study implied that GPT2 plays a role in sugar sensing either directly or indirectly by affecting the balance of metabolites among cellular compartments [62]. GPT connection with TPC and SWS - the two variables associated with this SNP - is unclear.

The functional annotation of the homologous sequence to that containing SNP ID TP15810 revealed a phospholipid-diacylglycerol acyltransferase (PDAT)*.* PDAT1 is a critical enzyme involved in triacylglycerol (TAG) synthesis [63], a high-energy compound. In plants, TAGs are generally synthesized on specialized cells of developing seeds, fruit, and pollen; and they are stored in lipid droplets, which are mobile during active periods of metabolism [64, 65]. Aging and stress factors such as freezing and desiccation can induce accumulation of TAGs in plant tissues [66–69] but the role of PDAT on this accumulation was not reported or was unknown since other enzymes like those of the Kennedy pathway can also lead to TAG biosynthesis. The connection between PDAT and the environmental variables that were associated to SNP ID TP15810 (SWp, MAP, and MAR) is therefore not evident.

The SNP ID TP69971 is located in a protein 100% identical to a segment (20 aminoacids) of ferredoxin--NADP(+) reductase in *Methylosinus, Methylocystis,* and *Paramesorhizobium.* These organisms are bacteria within the order Rhizobiales, an order known to contain nitrogen fixing bacteria in symbiotic relationship with plant roots [70] and genera like *Agrobacterium* known to have the capacity of transferring their DNA to their host plants [71]. We therefore, hypothesize that this SNP-containing locus might represent an old rhizobial DNA transfer to *V. vitis-idaea*. Ferredoxin—NADP(+) reductase (FNR) is an enzyme that participates in photosynthesis, but in non-photosynthetic organisms like bacteria, it provides reduced ferredoxin to other metabolic pathways including nitrogen fixation, terpenoid biosynthesis, steroid metabolism, oxidative stress response, and iron-sulfur protein biogenesis [72–74]. The link between this enzyme and the water-related environmental variables (MAP, SWp, SWS) is uncertain especially because plant dehydration did not induce significant changes in FNR activity in wheat [75].

A protein similar to a DEAD-box ATP dependent RNA helicase was annotated for an homologous sequence to that containing SNP ID TP45867. The DEAD-box RNA helicase family plays a role in DNA and RNA metabolism such as replication, repair, recombination, transcription, translation, ribosome biogenesis and splicing [76, 77]. These enzymes act in the growth and development of plants under stress by regulating stress-induced transcription and translation [78, 79]. For example, the overexpression of a gene in this family was associated with disease resistance and tolerance to oxidative stress [80, 81]. DEAD-box RNA helicases can regulate the function of transcripts involved in salt tolerance [82] and cold stress [81]. The role of these enzymes on alkalinity stress (SWp and SWS were the correlated variables), however, is unknown.

SNP ID TP29832 was associated with MAP and AC and the most significant blastx hits correspond to a chloroplast RNA binding protein 31B, also known as chloroplast ribonucleoprotein CP31 (cpRNP CP31). cpRNPs are nuclear encoded, highly abundant RNA binding proteins involved in chloroplast RNA processing and stabilization [83]. They react to external and internal signals, particularly light, but also cold stress by influencing multiple chloroplast RNA processing steps [84, 85]. It has also been shown that all or most cpRNP RNAs were repressed following attach by *Pseudomonas*, deprivation of nitrate, hypoxia or osmotic stress [84]. The role of cpRNPs on drought tolerance or the synthesis of phenolics is unknown.

The most significant blastx hits (96% identical) for SNP ID TP3574 corresponded to a mitochondrial pentatricopeptide repeat (PPR) protein. PPR proteins belong to a large gene family of sequence-specific RNA-binding proteins that regulate gene expression at the post-transcriptional level mainly in organelles but also in the nucleus [86]. Their functions comprise a wide range of physiological and developmental processes including cytoplasmic male sterility, photosynthesis, respiration and embryogenesis [86, 87]. Few functional studies of PPR proteins in plants have revealed a biotic and abiotic stress response. For example, they can respond to salt, abscisic acid, oxidative stress, and pathogen infection [86, 88, 89]. More importantly for our case study, some PPR proteins in maize, sugarcane and *Arabidopsis* were upregulated or downregulated in response to drought stress [90, 91]. TP3574 therefore deserves further exploration in light of its association with runoff.

A protein similar to a pre-mRNA splicing factor ATP dependent RNA helicase from the DEAH box family was annotated for an homologous sequence to that containing SNP ID TP60073. As with all other RNA helicases, the DEAH box subfamily plays a crucial role in plant growth, development, and stress response. Since TP60073 was associated with MAT, the following examples of temperature dependent control of gene expression by DEAH-box proteins motivates further exploration of this locus. A DEAH-box protein in *Arabidopsis* was found to regulate the efficiency of pre-mRNA splicing, and mutants of this gene showed severe defects in hypocotyl dedifferentiation and de novo meristem formation in tissue culture under high temperature [92]. In yeast, a DEAH-box RNA helicase mutant showed cold sensitive growth defects and impaired RNA unwinding activity in vitro [93].

SNP ID TP14763 was associated with TPC and the most significant blastx hits correspond to proteins containing a Myeloblastosis (MYB) DNA-binding domain. MYB is a large gene family that controls several processes like responses to biotic and abiotic stress, nutrient deficiency, hormone responses, trichome development, cellular proliferation and differentiation, cell shape and petal morphogenesis, primary and secondary metabolism, and defense [94]. Most notably for our study, several MYB transcription factors (TFs) have been reported to regulate the phenylpropanoid pathway, which produces several important phenolic compounds such as flavonoids [95–97]. In *Vaccinium* species such as bilberry, highbush blueberry and bog bilberry, potential R2R3 MYB genes involved in flavonoid biosynthesis have been found [98–102]. This SNP-bearing DNA region is worth of more in depth studies on the genetic changes related with different phenolic compound phenotypes. TFs are good targets for modifying complex traits in plants, thus we can expect that a technology based on TFs could lead the future of successful biotechnology crops [94]. The advent of transcriptome and genome databases for some *Vaccinium* species [99–101, 103–105] has revealed different families of TFs with potential roles in flavonoid biosynthesis. These databases, including ours, constitute an important resource to understand the signaling network involved in the regulation of these compounds’ synthesis.

Antioxidant properties and phenolic compounds have been measured in some European and North American cultivars (e.g. [9, 106]). Overall, the antioxidant properties, total phenolic, flavonoids, and anthocyanin content of these cultivars are lower than those of wild clones, which have been explained by phenology-related physiological differences between them. Cultivars do not show a significant variation of antioxidant activity (<50 GAE mg/g FW), whereas wild clones can vary more (125–200 GAE mg/g FW) [9]. North American cultivars can vary significantly with respect to total anthocyanin, total phenolic, and total tannin content [106]. Cultivars must be added to this genomic assay for a more comprehensive discovery of health-associated SNPs. For these markers to be used for lingonberry breeding, the differential expression of these SNP-containing genes should be first tested in response to environmental conditions in order to better understand their regulation mechanism. The heritability mode of these health-associated SNPs should also be investigated. A genetic linkage map should be constructed in a standard reference population for identifying markers that are close to the target genes associated with healthy traits. As the success of a breeding program depends on how closely associated these markers are to the healthy traits, other methods to achieve this could include quantitative trait loci analysis, association mapping, classical mutant analysis, and bulk segregant analysis [107].

We acknowledge the current discussion on the utility of restriction site associated DNA sequencing such as RADseq or GBS to detect loci under selection [108–110] and recognize that very likely there are still undetected potential adaptive loci in *V. vitis-idaea*. While accounting for the extent of linkage disequilibrium in a study system is an important consideration, the alternative methods proposed by Lowry et al. [108] – transcriptome sequencing, exon capture, whole genome sequencing and pool-seq – are also subject to equivalent limitations and require a larger genomic resource investment which is not available for many non-model plant species such as *V. vitis-idaea*. While the 132 putative SNPs identified in this study could be useful for the selection of genotypes with desirable climatic adaptations for breeding in a region with similar environmental conditions, further studies are needed to identify SNPs associated to abiotic and biotic stress. Finally, empirical validation of SNPs and the function of the annotated genes are required to corroborate the identified loci as adaptive.

## Conclusions

This study generated the first published genomic resource for *V. vitis-idaea*, and highlighted promising functional markers for molecular breeding in this species. Further studies are needed to validate the identified 132 putative loci associated to environmental variables and phenotypic traits, and to decipher the molecular basis of their role in local adaptation.

## Additional files

Additional file 1: Table S1. Sampled *V. vitis-idaea* sites in Newfoundland and Labrador, Canada. Each site is coded by ecoregion, mean annual temperature, mean summer temperature, mean annual precipitation, mean annual runoff, surface water pH, surface water sensitivity to acid rain, and proximity to the coast (Government of Newfoundland and Labrador 1992, and Alam et al. 2016). **Table S2.** Number of methods (LFMM, SamBada and Chi^2) that predicted a significant association (p < 0.01) between each SNP and biochemical/environmental variable. SNPs are the ones inferred to be significantly associated with a variable by at least two methods. **Table S3.** Number of SNPs in common between each pair of biochemical/environmental variables. Only SNPs inferred to be significantly associated with at least one variable by at least two methods are considered. **Table S4.** Moran.I test results per variable. **Table S5.** Data visualized in Fig. 4 Top. **Table S6.** Data visualized in Fig. 4 Bottom. (XLS 62 kb)

Additional file 2: PCoA plots. (PDF 104 kb)

## Abbreviations

AC: Antioxidant capacity
GBS: Genotyping-by-sequencing
MAP: Mean annual precipitation
MAR: Mean annual runoff
MAT: Mean annual temperature
MST: Mean summer temperature
NR: Non redundant
SNP: Single nucleotide polymorphism
SWp: Surface water pH
SWS: Surface water sensitivity to acid rain
TPC: Total phenolic content
