# Supplementary material for: Genetic variation associated with healthy traits and environmental conditions in Vaccinium vitis-idaea
Source: BMC Genomics. 2018 Jan 2;19:4. doi: 10.1186/s12864-017-4396-9 (PMC5748963; doi:10.1186/s12864-017-4396-9)
Supplement: Supplementary file 2 — PCoA plots. (PDF 104 kb) [file 12864_2017_4396_MOESM2_ESM.pdf]

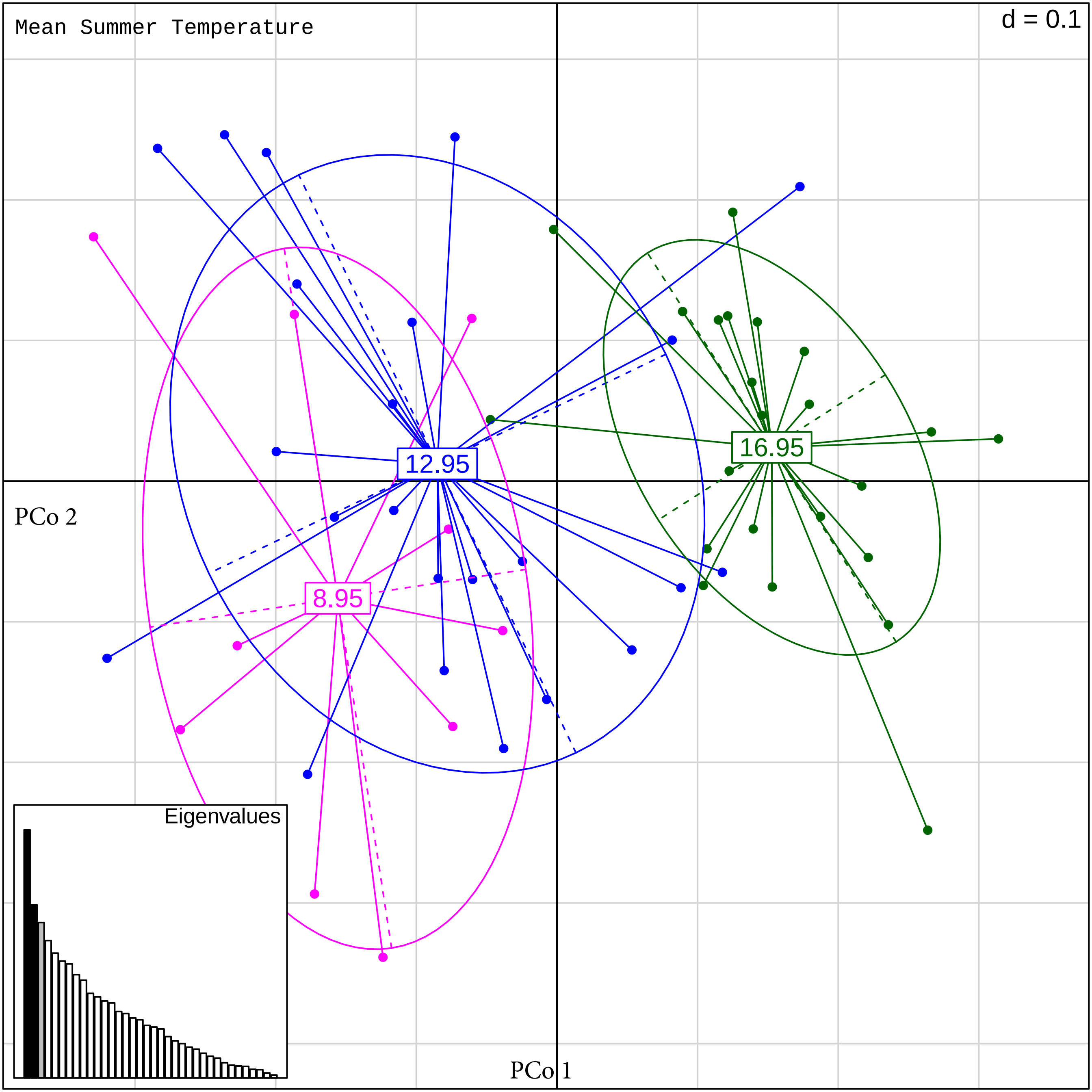
Mean Summer Temperature
d = 0.1
12.95
16.95
8.95
PCo 2
PCo 1
Eigenvalues

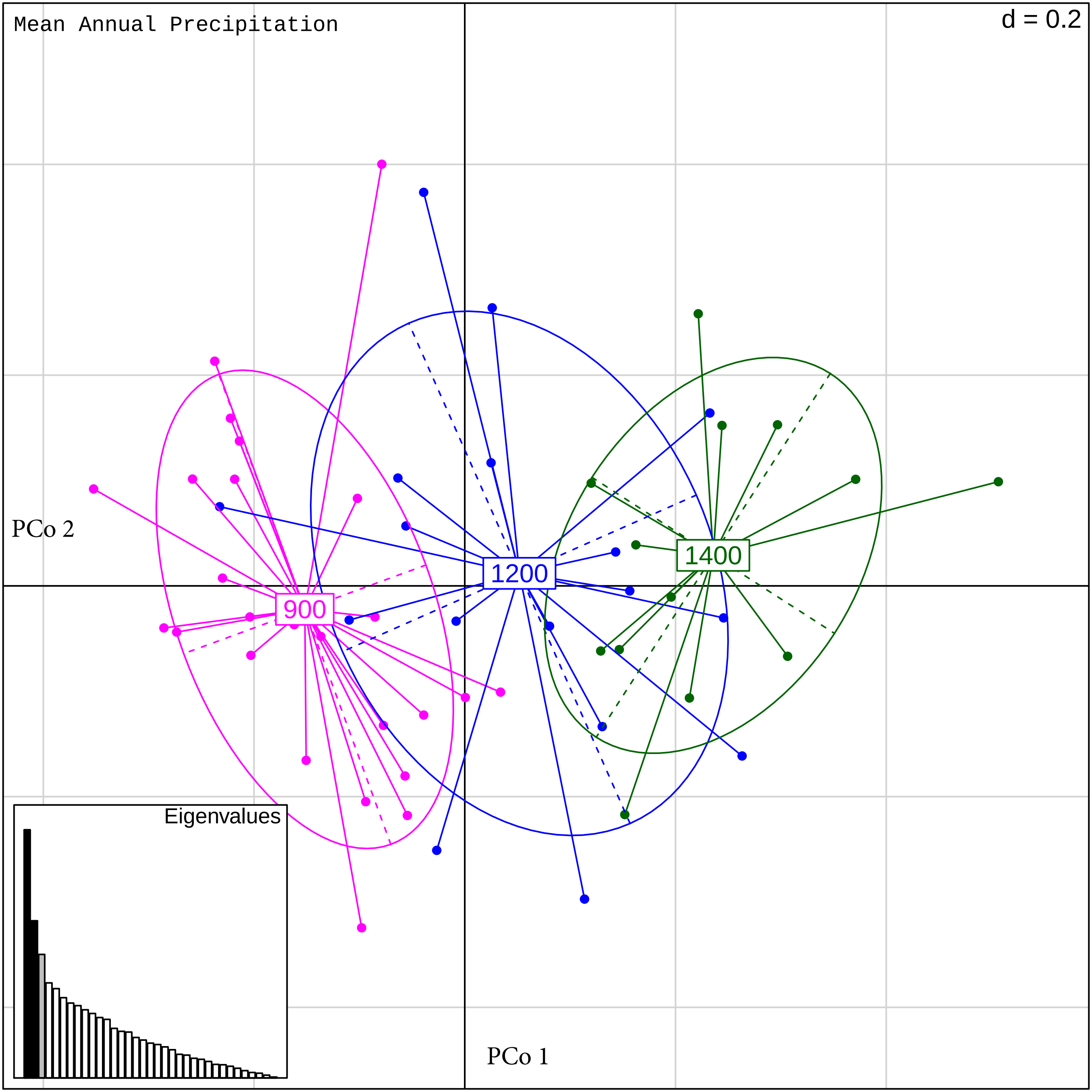

Mean Annual Precipitation
d = 0.2
PCo 2
PCo 1
900
1200
1400
Eigenvalues

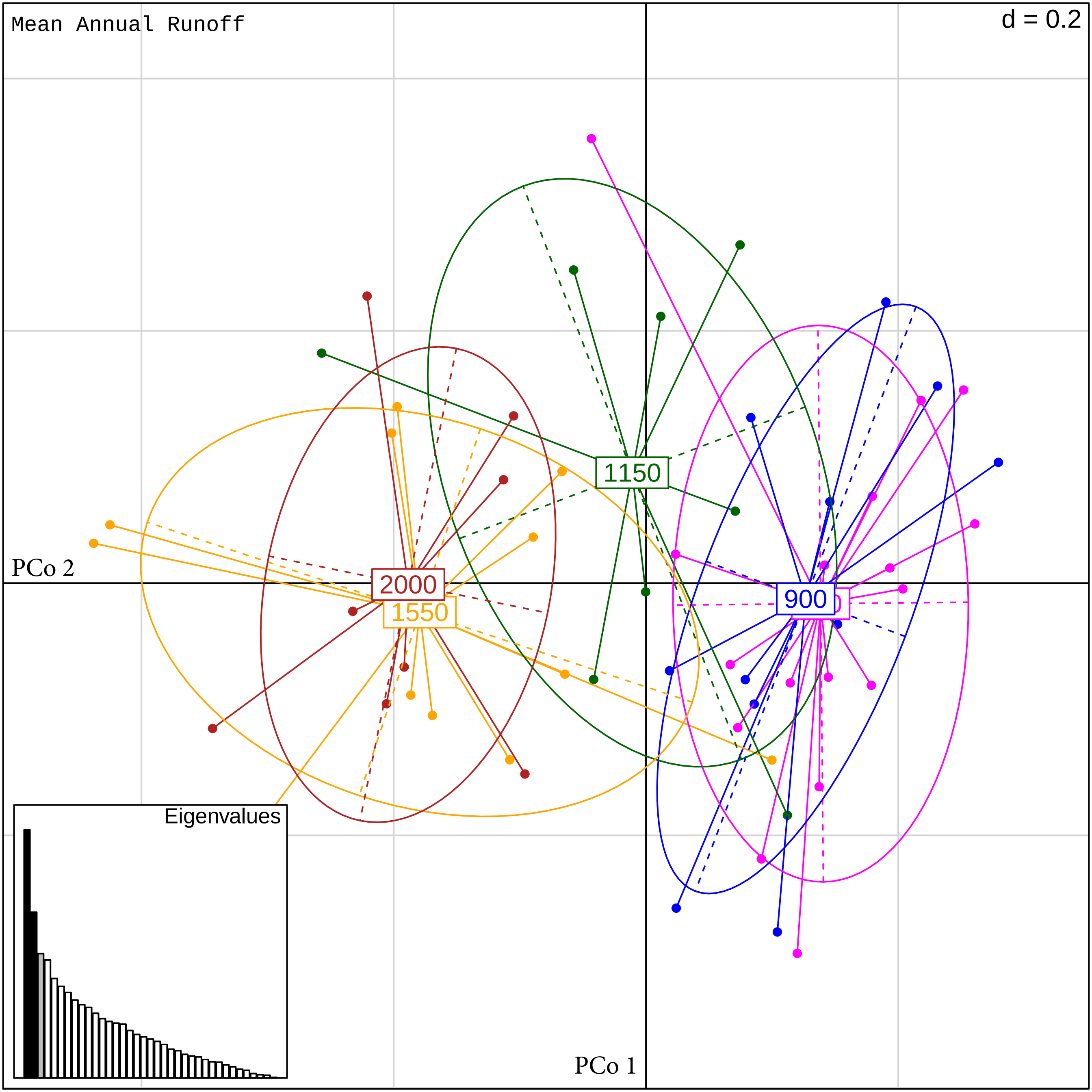

Mean Annual Runoff
d = 0.2
1150
2000
1550
900
PCo 2
PCo 1
Eigenvalues

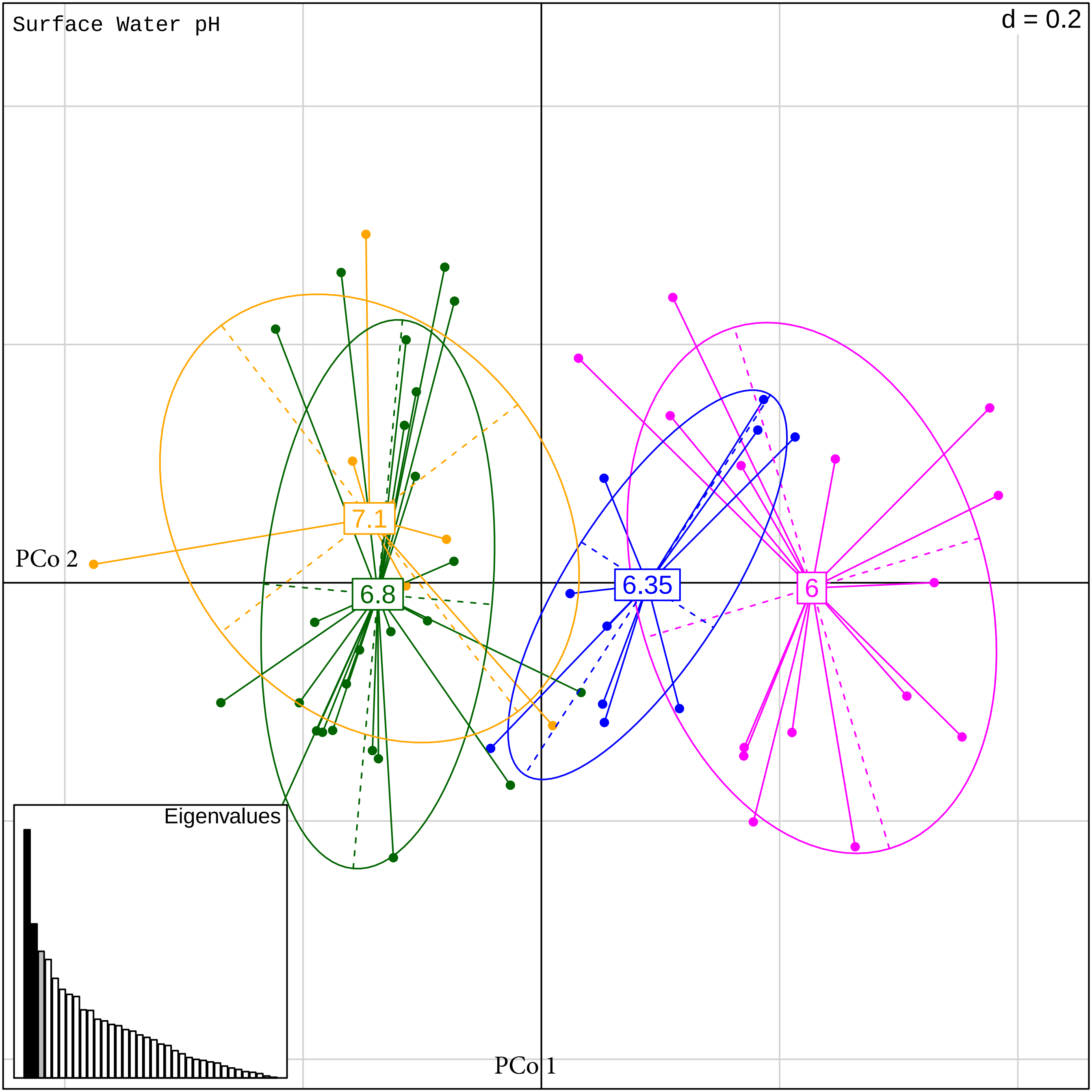

Surface Water pH
d = 0.2
PCo 2
PCo 1
7.1
6.8
6.35
6
Eigenvalues

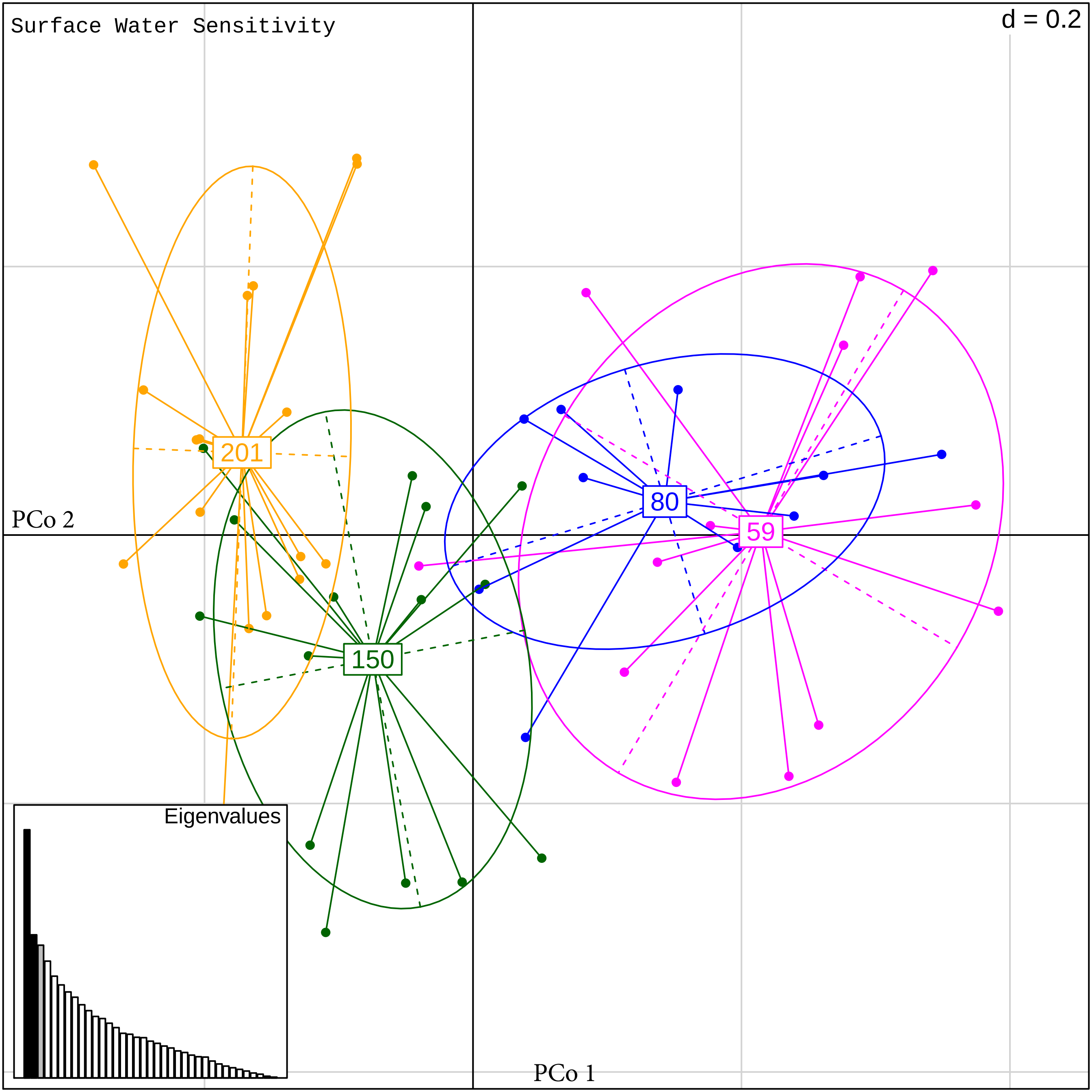
Surface Water Sensitivity
d = 0.2
PCo 2
PCo 1
Eigenvalues
201
150
80
59

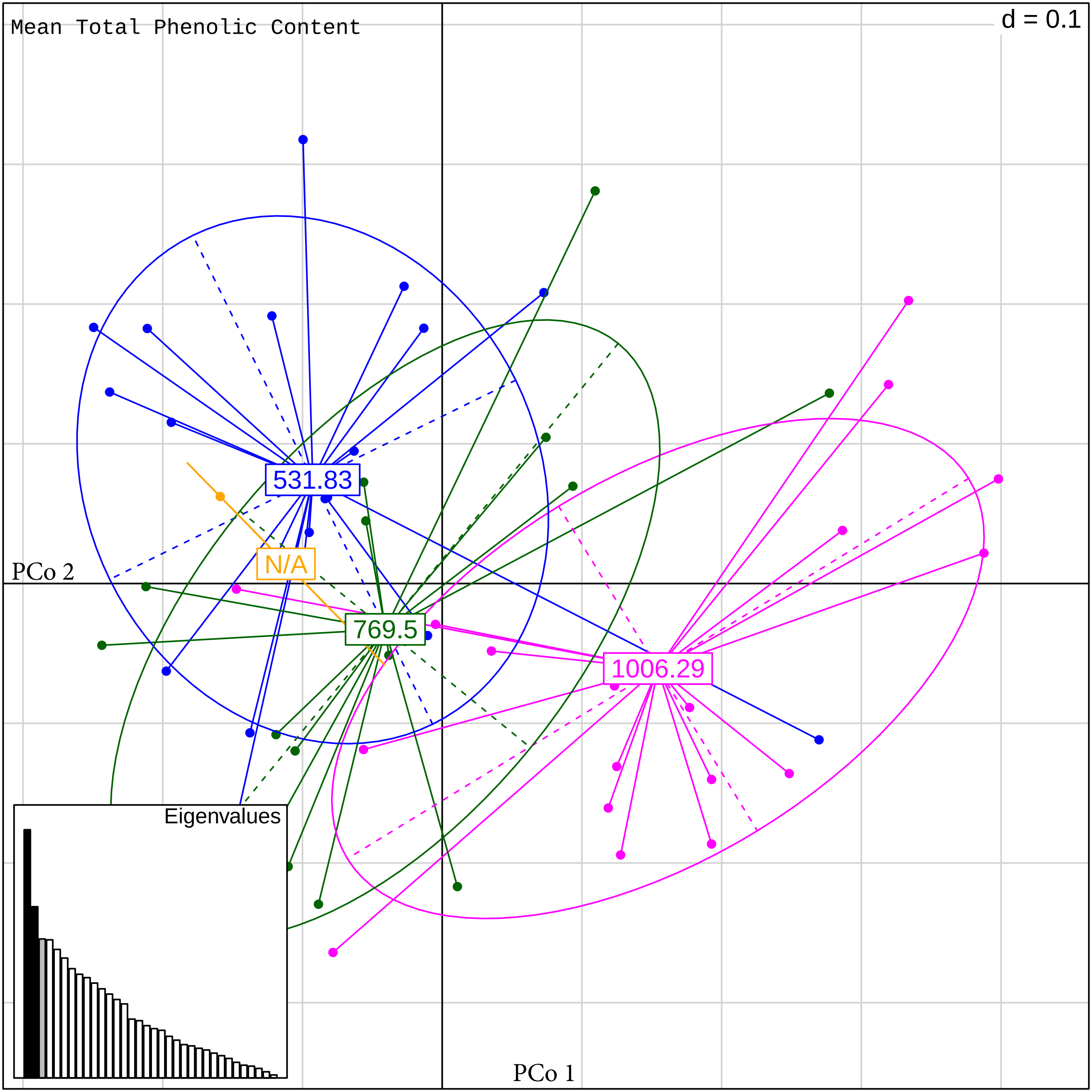

Mean Total Phenolic Content
d = 0.1
531.83
N/A
769.5
1006.29
PCo 2
PCo 1
Eigenvalues
